# Cholesterol profile in women with premature menopause after risk reducing salpingo-oophorectomy

**DOI:** 10.1007/s10689-018-0091-5

**Published:** 2018-06-07

**Authors:** Natalia Teixeira, Marian J. Mourits, Jan C. Oosterwijk, Ingrid E. Fakkert, Anthony R. Absalom, Stephan J. L. Bakker, Peter van der Meer, Geertruida H. de Bock

**Affiliations:** 1 Department of Epidemiology, University Medical Center Groningen, University of Groningen, Hanzeplein 1, 9713GZ Groningen, The Netherlands; 2 Department of Gynaecologic Oncology, University Medical Center Groningen, University of Groningen, Groningen, The Netherlands; 3 Department of Clinical Genetics, University Medical Center Groningen, University of Groningen, Groningen, The Netherlands; 4 Department of Anesthesiology, University Medical Center Groningen, University of Groningen, Groningen, The Netherlands; 5 Department of Internal Medicine, University Medical Center Groningen, University of Groningen, Groningen, The Netherlands; 6 Department of Cardiology, University Medical Center Groningen, University of Groningen, Groningen, The Netherlands

**Keywords:** *BRCA1*, *BRCA2*, Risk reducing salpingo-oophorectomy, Cholesterol, Dyslipidemia

## Abstract

This cross-sectional study aimed to investigate the effect of premenopausal risk reducing salpingo-oophorectomy (RRSO) on the cholesterol profile of women at increased ovarian cancer risk and to assess possible effects of age at and time since RRSO. We included 207 women who underwent RRSO before menopausal age (52 years) attending the family cancer clinic of an academic hospital and 828 age-matched women from a general population cohort (PREVEND). Participants filled out a questionnaire on socio-demographic characteristics, lifestyle and medical history, had anthropometric measurements and provided blood samples for assessment of serum levels of total cholesterol, HDL-cholesterol and non-HDL-cholesterol. The correlation between RRSO and cholesterol profile was assessed with logistic regression. Furthermore, subgroup analyses were performed to explore a possible effect of age at and time since RRSO. At a median time of 5.9 years (range 2.3–25.2) after surgery, RRSO was associated with low (< 60 mg/dl) HDL-cholesterol (OR 9.74, 95% CI 5.19–18.26) and high (≥ 160 mg/dl) non-HDL-cholesterol (OR 1.85, 95% CI 1.21–2.82) when adjusting for body mass index, hormone therapy, participation on sports and previous chemotherapy. The observed association was not dependent on age or time since RRSO. The RRSO group had less smokers (19.3 vs. 25.8%) and more participation on sports (45.4 vs. 22.0%). Our results suggest that RRSO is associated with a more atherogenic cholesterol profile, despite a lower prevalence of smoking and higher prevalence of participation on sports as compared to controls. This observation can be useful for physicians involved in the counselling and follow-up of women having RRSO.

## Introduction

Risk reducing salpingo-oophorectomy (RRSO) is the most effective strategy for preventing ovarian cancer (OC) in *BRCA1*/*2* germline mutation carriers, reducing OC risk up to 96% [1, 2] RRSO is currently recommended to these women after child bearing is complete, at age 35–40 (*BRCA1*) and 40–45 years (*BRCA2*). At this age the majority of women are premenopausal.

The decrease in sex hormone levels that occurs during menopausal transition through natural menopause affects body fat distribution and lipid metabolism [3] Postmenopausal status is associated with higher proportions of central fat, an increased risk of metabolic syndrome [4] and a more atherogenic lipid profile (higher levels of total, LDL-cholesterol [LDL-C] and non-HDL-cholesterol [non-HDL-C], and lower levels of HDL-Cholesterol [HDL-C]) [5–8], which is associated with the development and progression of atherosclerosis and an increased risk of cardiovascular disease (CVD) in the general population [9].

Bilateral oophorectomy causes an abrupt reduction in ovarian hormones, contrasting with natural menopause, a gradual process in which the ovaries continue to produce testosterone and androstenedione that can peripherally be aromatized to estrogen [10]. Despite its clear benefits for cancer prevention, RRSO has been associated with several non-cancer outcomes [11]. Pre-menopausal bilateral oophorectomy is associated with increased total cholesterol and reduced HDL-C (i.e. a more atherogenic lipid profile) [8, 12], a higher prevalence of metabolic syndrome and an increased risk of CVD and cardiovascular mortality when compared to natural menopause [13–17]. The impact of bilateral oophorectomy when performed with the specific purpose of reducing OC risk on the cardiovascular risk profile of women from hereditary breast and ovarian cancer families has not been extensively investigated yet. An association between RRSO and an increased risk of metabolic syndrome has been previously reported [18]. However, a recent study observed that women at increased risk of OC who underwent RRSO, presented a more favorable CVD risk profile than age-matched women [19].

Because the main goal of RRSO is to reduce morbidity and improve life expectancy in women with an increased cancer risk but who are otherwise healthy, it is important to investigate the potential adverse effects of RRSO, in order to provide women with individualized counseling and additional screening for non-oncological diseases when indicated. The aim of this study was to investigate the effect of RRSO on the cholesterol profile of women with an increased OC risk as compared to age-matched women who did not have oophorectomy.

## Patients and methods

### Patients

Surgical records of all women seen at the family cancer clinic of the University Medical Center Groningen with either a *BRCA1*/*2* mutation or a strong family history of breast/ovarian cancer were reviewed. We identified all women who had RRSO before the age of 52 years (mean menopausal age in the Netherlands) [20], with a follow-up period of at least two years after surgery. Between February 2011 and May 2012, these women were invited to participate in this study. In total, 254 women were invited, of whom 212 agreed to participate. Participants visited the outpatient clinic, filled out a questionnaire on socio-demographic characteristics, medical history, hormonal exposure [including use of systemic hormone replacement therapy (HRT)] and lifestyle, provided anthropometric measures and a blood sample, and had a bone mineral density measurement. Results on the association between RRSO and bone-related outcomes are available elsewhere [21, 22]. For the current study, patients who reported CVD before RRSO (N = 2) or from whom a blood sample was not available for analyses (N = 3) were excluded, and 207 remained for analyses. This study was considered extended standard care by the institutional ethics review board. All participants provided written informed consent.

### Age-matched women

Data from an age-matched group of women were acquired from the database of the PREVEND (Prevention of REnal and Vascular ENdstage Disease) study. In that study, all inhabitants of the city of Groningen aged between 28 and 75 years were invited to send a urine sample and to fill out a questionnaire on socio-demographic characteristics, hormonal exposure (including use of systemic HRT), medical history and lifestyle. Subjects with a urinary albumin concentration ≥ 10 mg/L and a randomly selected group of subjects with urinary albumin < 10 mg/L were invited to participate in the study. Participants were followed-up at regular time intervals (every 3–4 years) with anthropometric measurements, blood analysis, and a questionnaire on demographic characteristics, general health and lifestyle factors. Details of this cohort are described elsewhere [23]. Of the 8592 subjects enrolled, we selected all women who attended the third follow-up visit to the study clinic, between June 2003 and February 2006 (N = 2890). Women who reported hysterectomy or oophorectomy and/or a previous myocardial infarction (N = 700) were excluded. From the remaining 2190 women, we randomly allocated 4 age-matched women per study subject.

### Blood analysis

All individuals from both the RRSO and age-matched group provided a blood sample. Women from the age-matched group were asked to fast before providing a blood sample, while women from the RRSO group were not. For the RRSO group, total cholesterol, and HDL-C were measured with enzymatic methods (Roche/Hitachi modular analytics); for the age-matched control women, total cholesterol was estimated by a dry chemistry method (Eastman Kodak, Rochester, New York) and HDL-C was measured by a homogeneous method (direct HDL, Aeroset System; Abbott Laboratories, Abbott Park, Illinois). Non-HDL-C was calculated based on total cholesterol and HDL-C.

### Statistical analyses

Characteristics of the RRSO group and the age-matched women were described. Differences between groups were assessed with t-tests or Mann–Whitney’s test for continuous variables and with Chi^2^ or Fisher’s exact tests for categorical variables.

The main outcome-measures of this study were serum levels of total cholesterol, non-HDL-C and HDL-C. Total cholesterol was classified as high when ≥ 200 mg/dl, non-HDL-C was classified as high when ≥ 160 mg/dl and HDL-C was classified as low when < 60 mg/dl. We chose to classify cholesterol levels in this dichotomic manner (altered or not) in order to investigate whether RRSO was associated with differences on lipid profile that could lead to changes in patient management. Univariate logistic regression was applied to assess the correlation between factors covered by the questionnaire and the evaluated outcomes. The correlation between RRSO and total cholesterol (classified as normal vs. high), non-HDL-C (classified as normal vs. high) and HDL-C levels (classified as normal vs. low) was assessed with multivariate logistic regression, adjusting for possible confounders.

To explore potential differences on the impact on cholesterol profile of RRSO performed at an earlier age (< 45 years) and RRSO performed at a later age (≥ 45 years), a subgroup analysis was performed, stratifying women from the RRSO group according to age at surgery (< 45 or ≥ 45 years) and comparing women in each group to their respective controls. The cut-off age of 45 years was chosen based on a previous report [17]. To explore a possible effect of duration of hormonal depletion on total cholesterol, non-HDL-C and HDL-C levels, another subgroup analysis was performed, stratifying women from the RRSO group according to the time interval between RRSO and follow-up assessment (< 5 vs. ≥ 5 years) and comparing each group to their respective controls. All previous tests were repeated in both subgroup analyses, comparing each subgroup with their respective age-matched controls.

Because treatment for breast cancer and HRT may impact cardiovascular health, sensitivity analyses were performed to investigate the potential impact of these factors in our results. In a first analysis all women from the RRSO group with a history of breast cancer and their respective controls were excluded. For the second sensitivity analysis all women from the RRSO group who reported ever use of HRT and their respective controls were excluded. All tests previously performed were repeated in these subsets of women. To further investigate a possible impact of HRT on cholesterol profile, another sensitivity analysis was performed including only women from the RRSO group, applying logistic regression models to investigate the association between HRT use and cholesterol profile.

The association between the factors assessed and the outcomes was described with odds ratios (ORs) and 95% confidence intervals (95% CI), and p-values ≤ 0.05 were considered statistically significant. Random allocation of age-matched women was performed with R-Studio Version 0.98.1091 and statistical analyses were performed with IBM SPSS version 20.0 (IBM Corp., NY-USA).

## Results

### Study population

In total 207 women who had RRSO at premenopausal age and 828 age-matched women were included, at a mean age of 49.0 years (SD: 6.2). The mean age at RRSO was 42.7 years (SD: 5.3) and the median time interval between RRSO and assessment was 5.9 years (range 2.3–25.2).

Women from the RRSO group had significantly higher BMI than age-matched women (median 25.9 vs. 24.9 kg/m^2^). A significantly lower proportion of women in the RRSO group reported ever smoking (61.4 vs. 71.9%), while a larger proportion of women from the RRSO group reported regular participation on sports (63.3 vs. 47.9%), ever use of oral contraceptives (94.7 vs. 33.5%), HRT at any age (48.3 vs. 6.4%), and paid work (87.2 vs. 63.0%), compared to the age-matched women. A total of 77 (37.2%) women in the RRSO group had a history of cancer, compared to 41 (5.0%) of the age-matched women (Table 1).

**Table 1:** Demographic, health and lifestyle characteristics of women after RRSO and age-matched women

	Women after RRSO (n = 207)	Age-matched women (n = 828)	*p-*Value
Mean age in years (SD)	49.0 (6.2)	49.0 (6.2)	1.00
Mean age at RRSO in years (SD)	42.7 (5.3)	–	–
Median interval since RRSO in years (range)	5.9 (2.3–25.2)	–	–
Median BMI in kg/m^2^ (range)	25.9 (18.1–54.1)	24.9 (16.8–61.0)	0.01
Diabetes (ever reported during FUP)^a^	5/207 (2.4%)	14/828 (1.7%)	0.56
Hypertension (ever reported during FUP)^a^	28/207 (13.5%)	152/828 (18.4%)	0.10
Dyslipidemia (ever reported during FUP)^a^	13/207 (6.3%)	50/828 (6.0%)	0.90
Smoking (ever)	127/207 (61.4%)	595/828 (71.9%)	< 0.01
Smoking (current)	40/207 (19.3%)	213/828 (25.8%)	0.054
Sport (any)	131/207 (63.3%)	396/826 (47.9%)	< 0.01
Sport (> 2/week)^b^	94/207 (45.4%)	182/826 (22.0%)	< 0.01
Ever use of oral contraceptives	196/207 (94.7%)	277/828 (33.5%)	< 0.01
Ever use of HRT	100/207 (48.3%)	53/828 (6.4%)	< 0.01
Post-menopausal status	207/207 (100%)	333/820 (40.6%)	< 0.01
Paid work	164/188 (87.2%)	517/820 (63.0%)	< 0.01
Cancer	77/207 (37.2%)	41/828 (5.0%)	< 0.01
Chemotherapy	57/207 (27.5%)	2/828 (0.2%)	< 0.01
Radiotherapy	48/207 (23.2%)	0/828 (0.0%)	< 0.01
*BRCA1*/*2* mutation	176/207 (85.0%)	–	–
Cholesterol levels			
Mean total cholesterol in mg/dl (SD)	213.5 (46.7)	208.7 (39.4)	0.14
Mean non-HDL-C in mg/dl (SD)	170.6 (49.1)	148.6 (39.1)	< 0.01
Mean HDL-C in mg/dl (SD)	42.8 (14.1)	59.9 (14.5)	< 0.01
High cholesterol (≥ 200 mg/dl)	130/207 (62.8%)	477/828 (57.6%)	0.18
High non-HDL-C (≥ 160 mg/dl)	120/207 (58.0%)	312/828 (37.7%)	< 0.01
Low HDL-C (< 60 mg/dl)	182/207 (87.9%)	446/828 (53.9%)	< 0.01

*RRSO* risk reducing salpingo-oophorectomy, *SD* standard deviation, *BMI* body mass index, *FUP* follow-up, *HRT* hormone therapy

^a^Based on self-reported information for RRSO group and on pharmacy records for the age-matched women group

^b^≥ 2 h/week for women who had RRSO and ≥ 2 times a week for age-matched women

### Total cholesterol

Women from the RRSO group presented higher levels of total cholesterol than age-matched women (213.5 vs. 208.7 mg/dl), although this difference was not significant. After adjusting for BMI, ever use of HRT, chemotherapy and practice of sports, no correlation between RRSO and high cholesterol levels was observed (Table 2).

**Table 2:** Logistic regression analyses of the association of RRSO and possible confounding factors

	High total cholesterol			High non-HDL-C			Low-HDL-C		
	OR	95% CI	*p-*Value	OR	95% CI	*p-*Value	OR	95% CI	*p-*Value
Univariate									
RRSO	1.24	0.91–1.70	0.18	2.28	1.67–3.11	< 0.01	6.24	4.02–9.68	< 0.01
BMI > 25 kg/m^2^	1.58	1.23–2.03	< 0.01	2.11	1.64–2.71	< 0.01	2.15	1.67–2.77	< 0.01
Use of HRT	1.31	0.91–1.86	0.14	1.87	1.32–2.64	< 0.01	2.26	1.53–3.36	< 0.01
Smoking (ever)	1.03	0.79–1.35	0.83	1.13	0.87–1.49	0.36	0.84	0.64–1.10	0.21
Sport	0.85	0.66–1.09	0.20	0.78	0.61–1.00	0.05	0.72	0.56–0.93	0.01
Paid work	0.84	0.64–1.10	0.21	0.89	0.68–1.16	0.38	1.06	0.81–1.38	0.69
Chemotherapy	1.65	0.93–2.91	0.08	2.29	1.34–3.93	< 0.01	2.66	1.40–5.08	< 0.01
Multivariate									
RRSO^a^	1.12	0.78–1.60	0.54	2.02	1.41–2.88	< 0.01	6.29	3.87–10.22	< 0.01
RRSO^b^	0.94	0.62–1.44	0.79	1.85	1.21–2.82	0.01	9.74	5.19–18.26	< 0.01

*RRSO* risk reducing salpingo-oophorectomy, *OR* odds ratio, *BMI* body mass index, *HRT* hormone therapy

^a^Adjusted for BMI and HRT

^b^Adjusted for BMI, HRT, chemotherapy and practice of sports

### Non-HDL-C

Women in the RRSO group presented significantly higher levels of non-HDL-C than age-matched women (170.6 vs. 148.6 mg/dl), and a higher proportion of women after RRSO presented high non-HDL-C compared to age-matched women (58.0 vs. 37.7%; Table 1). RRSO was significantly associated with high non-HDL-C levels (OR 1.85; 95% CI 1.21–2.82), even after adjusting for BMI, ever use of HRT, chemotherapy and practice of sports (Table 2).

### HDL-C

The RRSO group presented significantly lower HDL-C levels than age-matched women (42.8 vs. 59.9 mg/dl), as well as a higher proportion of women with low HDL-C (87.9 vs. 53.9%; Table 1). RRSO was significantly associated with low HDL-C levels (OR 9.74; 95% CI 5.19–18.26) when adjusting for BMI, ever use of HRT, chemotherapy and practice of sports (Table 2).

### Subgroup analyses

After stratifying the cohort according to age at surgery (< 45 and ≥ 45 years) similar results were observed. After adjusting for possible confounders (BMI, ever use of HRT, previous chemotherapy and practice of sports) no significant association between RRSO and high total cholesterol was observed neither for women who had RRSO < age 45 years (N = 137 women with RRSO and 548 age-matched women) nor for women who had RRSO ≥ age 45 years (N = 70 women with RRSO and 280 age-matched women). A significant correlation between RRSO and high non-HDL-C was observed for women who had RRSO ≥ age 45 years (OR 2.04, 95% CI 1.04–4.00) but not for those who had RRSO before that age. RRSO was significantly associated with low HDL-C levels both among women who had RRSO < age 45 years (OR 14.65, 95% CI 5.45–39.40) and among women who had RRSO ≥ age 45 years (OR 6.97, 95% CI 2.96–16.39; Table 3). When stratifying the cohort according to time interval between RRSO and assessment, the association between RRSO and HDL-C remained significant both for women who had RRSO < 5 years before assessment (N = 72 women with RRSO and 288 age-matched women; OR 8.45; 95% CI 2.51–28.40) and for those who had RRSO ≥ 5 years before assessment (N = 135 women with RRSO and 540 age-matched women; OR 9.48; 95% CI 4.47–20.09), while the association between RRSO and high non-HDL-C was only significant for women who had RRSO < 5 years before assessment (OR 3.30, 95% CI 1.41–7.74). No significant association between RRSO and total cholesterol levels was observed in either of these subgroups (Table 3).

**Table 3:** Univariate and multivariate logistic regression analyses of the association between RRSO and high total cholesterol and low HDL-C in different subgroups

	High total cholesterol				High non-HDL-C			Low HDL-C		
	OR	95% CI	*p-*Value		OR	95% CI	*p-*Value	OR	95% CI	*p-*Value
RRSO < age 45 (N = 137 RRSO women and 548 age-matched women)										
Univariate	1.32	0.90–1.92		0.16	2.66	1.81–3.89	< 0.01	7.49	4.13–13.59	< 0.01
Adjusted for BMI and HRT	0.92	0.56–1.54		0.76	1.86	1.11–3.11	0.02	6.99	3.34–14.61	< 0.01
Adjusted for multiple factors^a^	0.64	0.34–1.21		0.17	1.39	0.74–2.59	0.30	14.65	5.45–39.40	< 0.01
RRSO ≥ age 45 (N = 70 RRSO women and 280 age-matched women)										
Univariate	1.10	0.63–1.95		0.73	1.77	1.03–3.03	0.04	4.90	2.52–9.52	< 0.01
Adjusted for BMI and HRT	1.11	0.63–1.97		0.73	1.81	1.05–3.11	0.03	5.87	2.95–11.66	< 0.01
Adjusted for multiple factors^a^	1.15	0.57–2.32		0.70	2.04	1.04–4.00	0.04	6.97	2.96–16.39	< 0.01
RRSO < 5 years (N = 72 RRSO women and 288 age-matched women)										
Univariate	1.81	1.07–3.08		0.03	3.21	1.89–5.46	< 0.01	6.96	3.22–15.04	< 0.01
Adjusted for BMI and HRT	1.82	0.91–3.65		0.09	3.14	1.56–6.32	0.01	5.87	2.33–14.80	< 0.01
Adjusted for multiple factors^a^	1.61	0.70–3.70		0.27	3.30	1.41–7.74	< 0.01	8.45	2.51–28.40	0.01
RRSO ≥ 5 years (N = 135 RRSO women and 540 age-matched women)										
Univariate	1.01	0.68–1.49		0.97	1.93	1.32–2.83	0.01	5.90	3.45–10.08	< 0.01
Adjusted for BMI and HRT	0.92	0.60–1.41		0.71	1.70	1.12–2.60	0.01	6.06	3.41–10.78	< 0.01
Adjusted for multiple factors^a^	0.77	0.46–1.29		0.32	1.51	0.91–2.50	0.11	9.48	4.47–20.09	< 0.01
Including only women without a history of breast cancer (N = 130 RRSO women and 520 age-matched women)										
Univariate	1.33	0.90–1.98		0.16	2.67	1.80–3.96	< 0.01	9.35	4.92–17.74	< 0.01
Adjusted for BMI and HRT	1.27	0.75–2.14		0.38	2.10	1.25–3.54	< 0.01	6.90	3.27–14.59	< 0.01
Adjusted for multiple factors^a^	1.28	0.75–2.17		0.37	2.19	1.29–3.72	< 0.01	7.60	3.56–16.22	< 0.01
Excluding women with ever HRT use (N = 107 RRSO women and 428 age-matched women)										
Univariate	1.06	0.68–1.67		0.79	1.90	1.24–2.92	< 0.01	5.33	3.03–9.37	< 0.01
Adjusted for BMI and HRT	1.11	0.71–1.75		0.64	1.97	1.27–3.05	< 0.01	5.35	3.01–9.49	< 0.01
Adjusted for multiple factors^a^	1.00	0.56–1.77		0.99	1.81	1.04–3.17	0.04	5.91	2.76–12.63	< 0.01

*RRSO* risk reducing salpingo-oophorectomy, *OR* odds ratio, *BMI* body mass index, *HRT* hormone therapy

^a^BMI, HRT, chemotherapy and participation on sporting activities

On a sensitivity analysis excluding women from the RRSO group who reported previous breast cancer (N = 77) and their respective age-matched group (N = 308), the association between RRSO and high non-HDL-C and between RRSO and low HDL-C levels remained significant even after adjusting for possible confounders (OR 2.19; 95% CI 1.29–3.72 and OR 7.60; 95% CI 3.56–16.22, respectively), while no association was observed between RRSO and total cholesterol. When restricting the analyses to women from the RRSO group who declared never having used HRT (N = 107) and their respective age-matched women (N = 428) significant associations between RRSO and high non-HDL-C and between RRSO and low HDL-C levels were observed after adjusting for confounders (OR 1.81; 95% CI 1.04–3.17 and OR 5.91; 95% CI 2.76–12.63, respectively), while no association between RRSO and total cholesterol was observed (Table 3). When restricting the analysis to women from the RRSO group, no significant associations were observed between HRT use and total cholesterol, non-HDL-C or HDL-C.

## Discussion

In this cohort study including 207 women with RRSO and 828 age-matched women, it was observed that after a median follow-up time of 5.9 years, women in the RRSO group had significantly higher levels of non-HDL-C and lower levels of HDL-C than age-matched women without oophorectomy. After adjusting for BMI, ever use of HRT, previous chemotherapy and participation on sports, RRSO was still significantly associated with increased non-HDL-C (OR 1.85; 95% CI 1.21–2.82) and with decreased HDL-C (OR 9.74; 95% CI 5.19–18.26). The association and the strength of the association between RRSO and HDL-C were observed regardless of age at RRSO or the time-interval between RRSO and assessment.

Serum levels of HDL-C and non-HDL-C correlate with cardiovascular risk [9, 24] and it has been suggested that lipid assessment can be simplified to total cholesterol and HDL-C measurement [24], which are the lipid levels accounted for in the most widely used algorithms for assessing the risk of cardiovascular events [25, 26].

To the best of our knowledge, only two previous studies investigated the cardio-metabolic risk profile after RRSO in women from hereditary breast and ovarian cancer families, indicating that although RRSO was associated with a higher prevalence of metabolic syndrome [18], women who had RRSO presented an overall healthier cardiovascular risk profile than age-matched women [19]. Those studies also observed higher levels of physical activity and a lower proportion of smokers in the RRSO group. It is possible that this highly selected group of women with a hereditary high risk of OC who chose to go through RRSO, facing its potential consequences in order to reduce their cancer risk, has an increased awareness of their health risks and may have a healthier lifestyle than age-matched women [19]. In the present study, although a significantly higher proportion of women in the RRSO group reported regular practice of sports and a lower proportion reported smoking, they presented a more atherogenic lipid profile than age-matched women.

Possible explanations for the contrasting results observed in the present analyses and in one of the aforementioned studies [19] are that only 68% of women included in the former study had RRSO at age 52 or younger, while all women in the current study had RRSO before that age. It has been suggested that women who had an early oophorectomy are at an increased risk of cardiovascular disease when compared to women who had oophorectomy at later ages [14, 17, 27]. Hence it is plausible that RRSO performed at younger age has a more pronounced impact on lipid metabolism than when performed at older age (e.g. after age of natural menopause). To explore a possible impact of age at RRSO on the lipid profile, we performed subgroup analyses stratified according to age at surgery. Although the association between RRSO and high non-HDL-C was only significant for women who had RRSO ≥ age 45 years, a significant association between RRSO and lower HDL-C was observed both for women who had surgery < age 45 (OR 14.65, 95% CI 5.45–39.40) and ≥ age 45 (OR 6.97, 95% CI 2.96–16.39). Although these results do not confirm the hypothesis that early RRSO has a stronger impact on lipid profile than RRSO at later ages, the number of women included in each subgroup was limited, which was reflected by wide confidence intervals. This uncertainty in the estimates could explain the lack of a significant effect of early age at RRSO on its impact on lipid profile.

Furthermore, women included in the current study were on average younger than in the former study (mean age 48.7 vs. 54.4). It is possible that women who had RRSO only have a less favorable lipid profile when compared to premenopausal women, and once all women reach natural menopause, this difference can no longer be observed. To explore the possible impact of time interval after RRSO, an analysis stratified according to time interval between surgery and assessment was performed. The association between RRSO and non-HDL-C was only significant for women with < 5 years of follow-up. Nevertheless, RRSO was associated with lower HDL-C both in the group with follow-up < 5 years (OR 8.45; 95% CI 2.51–28.40), and ≥ 5 years (OR 9.48; 95% CI 4.47–20.09).

Strengths of this study are the inclusion of a control arm with age-matched women and that all women in the RRSO group had surgery before age 52 (mean age of natural menopause). Furthermore, age-matched women were selected from a cohort that covers the population of the same geographical area where the RRSO group was ascertained.

Limitations are that information on medical history and lifestyle was self-reported and collected in different time-frames for the RRSO (2011–2012) and control groups (2003–2006). However, there is no evidence that lifestyle and lipid profile varied significantly in the population under study during this period of time and therefore this should not have any impact on the results of this study. Moreover we did not have detailed information on age at start and duration of HRT use, and could not investigate its impact on the cholesterol profile in more detail. Although HRT use for prevention of cardiovascular disease is a historically controversial topic, recent studies show compelling evidence that HRT use after surgical menopause is associated with cardiovascular protection, especially when initiated immediately after surgery [28]. Other limitations are that only patients who were alive at the time of assessment were included and results might be influenced by survival bias. However, we assume this effect to be very small because only 10 of the women initially identified were deceased, none of them due to cardiac causes. Furthermore, information on other factors that might impact cardiovascular risk such as cholesterol levels previous to RRSO, diet, blood pressure, serum levels of LDL-C and triglycerides, as well as family history of cardiovascular disease was not available and could not be controlled for. The incidence of cardiovascular events was also not investigated, since such events might take a very long time to occur and its assessment would require a longer follow-up time. Furthermore, the power of subgroup analyses may have been limited by the modest sample size of each subgroup. It is also important to note that women from the RRSO group were not asked to fast before providing a blood sample, while the age-matched women were. This might have led to a small difference in total cholesterol concentrations, but the documented size of this effect is not large enough to explain the difference that we found [29, 30]. Another potential limitation is the fact that two different laboratory assessment techniques have been applied for measurement of circulating lipid concentrations. Tough it cannot be excluded that this explains differences, this is very unlikely, because in the Netherlands, important laboratory assays like those for total cholesterol and HDL-cholesterol are tightly controlled and differences between laboratories minimized by means of proficiency testing procedures coordinated by the Dutch Foundation for Quality Assessment in Medical Laboratories (SKML).

A significantly larger proportion of women in the RRSO group had cancer (37.2 vs. 5.0%) and received chemotherapy (27.5 vs. 0.2%) than in the group of age-matched women, and chemotherapy has been associated with metabolic changes [31, 32]. Since only two of the age-matched women had received chemotherapy, it was not possible to fully investigate its impact on our results. To account for differences between the study groups, we performed a sensitivity analysis, excluding all women who had cancer and their respective age-matched women, the associations between RRSO and high non-HDL-C and between RRSO and low HDL-C were still observed. Ideally, to explore the impact of RRSO on cholesterol profile, women who had RRSO should be compared to women at increased risk of OC who chose not to have RRSO. This would insure that baseline characteristics that could impact several aspects of their health were more comparable between both groups. However, RRSO uptake among women at increased risk of ovarian cancer is considerably high, which makes it difficult to assemble a sufficiently large group of women who chose not to have RRSO.

This study indicates that pre-menopausal RRSO is associated with higher serum levels of non-HDL-C, and lower serum levels of HDL-C. RRSO was associated with low HDL-C levels regardless of the age at which surgery was performed or the duration of hormonal depletion. The observed associations were found despite a healthier lifestyle and could not be attributed to previous chemotherapy or HRT use. When confirmed in further studies these results could have implications for the pre-operative counseling and for the long-term follow-up after RRSO, by raising awareness over the possibly more atherogenic cholesterol profile of these women. When longer follow-up times are available, it would be interesting to assess the incidence of cardiovascular events in women who had RRSO compared to age-matched women in a prospective study.

## Abbreviation

RRSO: Risk reducing salpingo-oophorectomy
